# Altered Functional Connectivity of the Nucleus Accumbens Network Between Deficit and Non-deficit Schizophrenia

**DOI:** 10.3389/fpsyt.2021.704631

**Published:** 2021-09-30

**Authors:** Chao Zhou, Chen Xue, Jiu Chen, Nousayhah Amdanee, Xiaowei Tang, Hongying Zhang, Fuquan Zhang, Xiangrong Zhang, Caiyi Zhang

**Affiliations:** ^1^Department of Geriatric Psychiatry, Affiliated Nanjing Brain Hospital, Nanjing Medical University, Nanjing, China; ^2^Department of Radiology, Affiliated Nanjing Brain Hospital, Nanjing Medical University, Nanjing, China; ^3^Institute of Neuropsychiatry, The Affiliated Brain Hospital of Nanjing Medical University, Nanjing, China; ^4^Institute of Brain Functional Imaging, Nanjing Medical University, Nanjing, China; ^5^Department of Psychiatry, Affiliated WuTaiShan Hospital of Medical College of Yangzhou University, Yangzhou, China; ^6^Department of Radiology, Subei People's Hospital of Jiangsu Province, Yangzhou University, Yangzhou, China; ^7^Department of Psychiatry, Affiliated Xuzhou Oriental Hospital of Xuzhou Medical University, Xuzhou, China

**Keywords:** deficit schizophrenia, functional connectivity, nucleus accumbens, resting-state fMRI, neurocognition

## Abstract

Deficit schizophrenia (DS), which is marked by stable negative symptoms, is regarded as a homogeneous subgroup of schizophrenia. While DS patients have structurally altered nucleus accumbens (NAcc) compared to non-deficit schizophrenia (NDS) patients and healthy individuals, the investigation of NAcc functional connectivity (FC) with negative symptoms and neurocognition could provide insights into the pathophysiology of schizophrenia. 58 DS, 93 NDS, and 113 healthy controls (HCs) underwent resting-state functional magnetic resonance (rsfMRI). The right and left NAcc were respectively used as seed points to construct the functional NAcc network in whole-brain FC analysis. ANCOVA compared the differences in NAcc network FC and partial correlation analysis explored the relationships between altered FC of NAcc, negative symptoms and neurocognition. Compared to HCs, both DS and NDS patients showed decreased FC between the left NAcc (LNAcc) and bilateral middle cingulate gyrus, and between the right NAcc (RNAcc) and right middle frontal gyrus (RMFG), as well as increased FC between bilateral NAcc and bilateral lingual gyrus. Moreover, the FC between the LNAcc and bilateral calcarine gyrus (CAL) was lower in the DS group compared to NDS patients. Correlation analysis indicated that FC value of LNAcc-CAL was negatively correlated to negative symptoms. Furthermore, aberrant FC values within the NAcc network were correlated with severity of clinical symptoms and neurocognitive impairments in DS and NDS patients. This study demonstrated abnormal patterns of FC in the NAcc network between DS and NDS. The presence of altered LNAcc-CAL FC might be involved in the pathogenesis of negative symptoms in schizophrenia.

## Introduction

The reward network is usually implicated in schizophrenia, especially in the regulation of negative symptoms (1–3). The nucleus accumbens (NAcc), which is a core node of the reward network, modulates information flow from the amygdaloid complex to the basal ganglia, mesolimbic dopaminergic regions, mediodorsal thalamus, and prefrontal cortex. NAcc also plays a prominent role in human cognitive, emotional, and psychomotor functions (4, 5).

Structural abnormalities of the NAcc were consistently demonstrated in schizophrenia. There were decreased right (6) and bilateral (7) NAcc volumes in first-episode psychosis subjects compared to healthy controls (HCs). Two meta-analyses with large sample size showed significant volume reductions of the NAcc in schizophrenia patients (8, 9). Another study indicated that there were lower NAcc volumes when antipsychotic medication therapy was discontinued in patients with schizophrenia (10). While previous neuroimaging studies of NAcc in schizophrenia were primarily focused on structural alterations, functional neuroimaging studies *via* functional magnetic resonance imaging (fMRI) analyses are scarce.

A fMRI study which utilized a word-image associative encoding task showed a signal change in the NAcc of patients with schizophrenia, which negatively correlated to the Physical Anhedonia Scale scores (11). Furthermore, altered functional connectivity (FC) between the NAcc and other brain regions, including the left temporal superior gyrus, cingulate gyri, and ventral tegmental area, was associated with the pathogenesis of hallucinations among patients with schizophrenia (12). Overall, the above-mentioned findings suggested the presence of aberrant changes in the NAcc of patients suffering from schizophrenia. However, whether the functional alterations of NAcc play a crucial role in negative symptoms in schizophrenia remain unclear.

Deficit schizophrenia (DS), which is characterized by primary and persistent negative symptoms during clinical stability periods, is regarded as a clinically homogeneous subgroup of schizophrenia (13–17). Previous studies have demonstrated that DS patients differed from non-deficit schizophrenia (NDS) patients in numerous clinical aspects, including risk factors, premorbid functioning, disease course, neurobiological correlates, and response to treatment (18–20). Neuroimaging studies have also revealed the presence of specific structural and functional alterations in brain regions and networks of DS patients compared to NDS patients (16, 21–23). Nevertheless, few studies focused on the NAcc network in DS patients. Only one structural neuroimaging study showed that DS patients had smaller left NAcc volumes compared to both NDS patients and HCs. Moreover, the lower left NAcc volumes significantly correlated with age and duration of illness (24). As a homogeneous subgroup of schizophrenia, studies on DS could provide better insight into the pathogenesis and development of schizophrenia, including the negative symptoms in schizophrenia. However, to date, no study has reported FC alterations of the NAcc network in DS patients via resting-state fMRI. Furthermore, the relationships between altered FC of the NAcc network and negative symptoms as well as neurocognition is still unclear.

In the present study, we conducted resting-state fMRI scans, clinical assessments and neurocognitive tests in order to investigate the functional characteristics of NAcc in DS patients. We set the right and left NAcc as two individual seed points to construct and compare the whole brain FC network of the two patient groups. Moreover, we investigated the relationship between altered FC with clinical and cognitive characteristics. We hypothesized that DS and NDS patients would exhibit convergent and divergent abnormal FC within the NAcc network. We also hypothesized that altered FC of the NAcc network would be associated with clinical symptoms, especially with negative symptoms, and neurocognitive deficits in DS and NDS patients.

## Methods and Materials

### Subjects

A total of 264 male participants, including 151 clinically stable schizophrenia patients (58 DS and 93 NDS) and 113 HCs, were enrolled in this study. All patients were enlisted at the inpatient department of the psychiatric rehabilitation unit of Yangzhou Wutaishan Hospital in Jiangsu Province, China while HCs were recruited via advertisements in the local community.

The eligibility criteria for patients included in the study are as follows: (1) a diagnosis of schizophrenia according to the Diagnostic and Statistical Manual of Mental Disorders (DSM)-IV and confirmed by a psychiatrist using the Chinese version of Structured Clinical Interview for DSM-IV (SCID-I) (25); (2) right-handed Han Chinese patients aged between 20 and 65 years; and (3) stabilized psychiatric symptoms with antipsychotic medications for at least 12 months prior to participation in the study. Patients with any of the following criteria were excluded: (1) severe comorbid conditions (i.e., head trauma, intellectual disability); (2) a history of substance abuse (e.g., alcohol and/or drugs); (3) previous physical therapies such as electroconvulsive therapy; and (4) contraindications for MRI.

Patients were diagnosed as either DS or NDS according to the Chinese version of Schedule for the Deficit Syndrome (SDS) (26). SDS evaluates the presence of deficit syndrome based on two criteria. The first criterion assesses the presence of any two of the following symptoms: restricted affect, diminished emotional range, poor speech, loss of interest, diminished sense of purpose, and reduced social drive. For the second criterion to be met, the symptoms should be at least moderately severe, persistent over 12 months, and not caused by a secondary condition (i.e., medication side effects, depression, paranoia, or anxiety).

The 113 male HCs included in this study were also right-handed, and matched for age. They were assessed *via* unstructured clinical interviews in order to exclude: (1) a history of organic brain disorders, intellectual disability, or severe head trauma; (2) a history of neurological or psychiatric illnesses; (3) a family history of psychiatric disorders in first degree relatives; (4) any serious physical disease; and (5) contraindications for MRI.

This study was approved by the Institutional Ethical Committee for Clinical Research of ZhongDa Hospital Affiliated to Southeast University. All participants provided written informed consent.

### Assessments of Clinical Symptoms and Neurocognition

Brief Psychiatric Rating Scale (BPRS), Scale for the Assessment of Negative Symptoms (SANS) and Scale for the Assessment of Positive Symptoms (SAPS) were utilized so as to evaluate the severity of negative and positive symptoms. The BPRS scale was divided into positive, negative, disorganized, and affect-syndromes, according to the results of the most comprehensive factor analysis of the 18-item BPRS (27).

Each subject completed eight standard neurocognitive tests, including the Digit Vigilance Test (DVT), the Animal Naming Test, the Controlled Oral Word Association Test (COWAT), a Block Design [Wechsler adult intelligence scale-Chinese Revision (WAIS-RC)], the Trail Making Test-A, -B (TMT-A, -B), the Stroop ColorWord Test, and the Spatial Processing Test. The raw scores obtained from each of these tests were transformed into *z*-scores. However, if the raw scores of any of the abovementioned test were inconsistent with the observed behavioral performance on the respective test, as with TMT-A/B, the scores were adjusted as reciprocal of the test value before transformation to *z*-scores so as to allow for uniformity in subsequent statistical analyses.

### MRI Data Acquisition

All subjects were scanned using a 3T magnetic resonance (MR) system (GE HDx, Chicago, IL) using an 8-channel phased array head coil at Subei Hospital of Jiangsu Province, China. Images were obtained through the use of a gradient recalled echo-echo planar imaging (GRE-EPI) sequence with the following parameters: repetition time (TR) = 2,000 ms, echo time (TE) = 25 ms, flip angle = 90°, slice number = 35, field of view (FOV) = 240 × 240 mm^2^, slice thickness = 4 mm (without a gap), matrix size = 64 × 64, voxel size = 4 × 4 × 4 mm^3^, and volume number = 240. During the MRI scan, all participants were asked to lie in the scanner with their eyes closed and their head firmly positioned inside the coil in order to minimize head motion. The resting functional MRI scan recording lasted 8 min.

### Imaging Pre-processing

The fMRI data was pre-processed using the Statistical Parametric Mapping 8 (SPM8) software in MATLAB (http://www.fil.ion.ucl.ac.uk/spm/software/SPM), released in 2016a (http://www.mathworks.com/products/matlab/). The first 10 images were excluded due to saturation effects and/or equilibrium magnetization. Echo planar images from each participant were then corrected for acquisition time delay among different slices and were realigned to the first volume for correction of head-motion. The resulting functional images were spatially normalized to the standard Montreal Neurological Institute (MNI) space through the use of a T1 image unified segmentation, and resampled into 3 mm isotropic voxels. For quality control, organic abnormality was examined and excluded using T1 weighted MRI. The images were then temporally band-pass filtered (0.01–0.10 Hz) after eliminating the linear trends of time courses in order to reduce the effect that emerged from low-frequency drifts and high-frequency noise. The nuisance signals, including six head motion parameters, cerebrospinal fluid signals, white matter signals, and global mean signals, were regressed from the data as corrected values. Finally, the images were smoothed using an 8-mm full width at half maximum Gaussian kernel. A scrubbing procedure was performed so as to reduce bias on the resting state (R)-fMRI signals induced by head motion artifacts. In brief, the root mean square deviation (dRMS) of the framewise displacement was calculated (28) between neighboring functional volumes within each subject. Next, the volume values were scrubbed using a dRMS over 0.5 mm along with the adjacent volume values (one back and two forward) for R-fMRI results of each subject.

### Network Construction

In the present study, the left and right NAcc were set as regions of interest (ROIs), as defined by the Wake Forest University Pick Atlas (29), and were used as seed points to construct the left and right functional connectivity maps of the NAcc network, respectively. Pearson correlation analysis was employed in order to obtain the correlation coefficient (*r*-value) of mean time series between the seed point and whole brain voxel for each participant. Fisher *Z* transformation was then conducted to convert the *r* value to a normally distributed *Z*-value. The numerical values obtained from altered FC regions were then extracted with the Resting-State fMRI Data Analysis Toolkit (REST) (http://resting-fmri.sourceforge.net/), using the altered regions as masks. Thereafter, FC values were converted to *Z*-values via Fisher *Z* transformation and subsequently used for the further correlation analysis.

### Statistical Analyses

Statistical analyses were carried out using the Statistical Package for the Social Sciences (SPSS) software version 19.0 (IBM, Armonk, NY). Chi-squared test, two-sample *t*-tests, and analysis of variance (ANOVA) were performed in order to compare demographic, clinical, and neurocognitive variables. Bonferroni test was utilized for *post-hoc* comparisons. Analysis of covariance (ANCOVA) was conducted using DPABI (a toolbox for Data Processing and Analysis for Brain Imaging, http://rfmri.org/dpabi) so as to determine FC alterations in the NAcc network among DS, NDS, and HC groups with age and education as covariates. In addition, when there was a significant group effect, *post-hoc* multiple comparisons with *t*-test were performed in order to detect the differences between each pair of groups. The 3dClustSim correction was performed for *post-hoc* multiple comparisons at voxel level (https://afni.nimh.nih.gov/pub/dist/doc/program_help/3dClustSim.html). The statistical threshold was set at *p* < 0.05 after correction. Partial correlation analysis was conducted using age, education, chlorpromazine equivalent, and illness duration as covariates in order to determine the relationship between aberrant FC, clinical features and neurocognitive assessments in both the DS and NDS groups. The statistically significant *p*-value was set as ≤ 0.05.

## Results

### Demographic and Clinical and Neurocognitive Features

The demographic characteristics of all participants are displayed in Table 1. Education level (ANOVA; *p* < 0.001) was significantly different among the three groups. *Post-hoc* comparisons indicated that both DS (*p* < 0.05) and NDS (*p* < 0.05) patients had lower education compared to HCs. There were no significant differences for age and tobacco usage among the three groups (ANOVA; *p* = 0.856; χ^2^ = 1.89, *p* = 0.388). Additionally, duration of illness, age of onset, and antipsychotic medicine dosage did not differ significantly between the two patient subgroups (*p* > 0.05).

**Table 1:** Demographic characteristics of DS, NDS, and HC.

**Variable**	**DS**	**NDS**	**HCs**	**χ2/*****t***/***F***
	**(***n*** = 58)**	**(***n*** = 93)**	**(***n*** = 113)**	**(***p***-value)**
Age (years)	51.50 ± 9.99	51.34 ± 8.84	50.81 ± 7.77	0.16 (0.856)
Education (years)	8.05 ± 2.78^*^	8.86 ± 2.62^*^	10.63 ± 2.48	22.30 (<0.001)
Duration of illness (years)	28.74 ± 8.42	27.77 ± 9.24		0.41 (0.523)
Age of onset (years)	22.72 ± 3.42	23.64 ± 5.02		1.49 (0.224)
Smoker/Non-smoker	26/32	32/61	47/66	1.89 (0.388)^a^
Dose of Antipsychotic, mg/d (chlorpromazine equivalent)	470.00 ± 263.17	503.28 ± 257.38		0.77 (0.445)

*Data is presented as mean ± standard deviation. Bonferroni correction was used for post-hoc comparisons*.

a *The P-value was obtained by using a chi-square test*.

* *Indicates the comparison between patients and HC*.

* *p < 0.05*.

The clinical and neurocognitive features of the two patient subgroups are presented in Table 2. In contrast to NDS patients, DS patients had significantly higher scores on the negative syndrome section of BPRS, total BPRS and total SANS (*p* < 0.001). However, there were no significant differences in positive, affect, and disorganized syndromes parts of BPRS or SAPS between the DS and NDS groups (*p* > 0.05). With regards to the neurocognitive characteristics (Table 2), *post-hoc* comparisons indicated that HCs performed better than both patient subgroups on all cognitive tests (*p* < 0.05). Furthermore, DS patients had poorer performance on DVT, TMT-A, Stroop color-only test, Stroop word-only test, ANT, and WAIS-RC (block design; *p* < 0.05) compared to NDS patients.

**Table 2:** Clinical and cognitive features of DS, NDS, and HC.

**Variable**	**DS**	**NDS**	**HCs**	**χ2/*****t***/***F***
	**(***n*** = 58)**	**(***n*** = 93)**	**(***n*** = 113)**	**(***p***-value)**
**BPRS**				
Positive syndrome	6.07 ± 1.13	6.30 ± 1.28		0.40 (0.531)
Negative syndrome	12.21 ± 2.16	7.40 ± 1.05		299.79 (<0.001)
Disorganization syndrome	6.48 ± 1.38	6.29 ± 0.93		0.27 (0.606)
affect	6.84 ± 1.17	6.91 ± 1.29		0.10 (0.757)
Total Scores	31.61 ± 3.31	26.91 ± 2.80		75.39 (<0.001)
**SANS**				
Total Scores	47.59 ± 12.08	28.89 ± 9.27		121.00 (<0.001)
**SAPS**				
Total scores	8.59 ± 3.48	9.73 ± 4.66		1.73 (0.190)
***Z*** **-scores of cognitive scales**				
Digit vigilance test	−0.69 ± 0.65^***^/^###^	0.14 ± 0.81^*^	0.80 ± 0.91	22.511 (<0.001)
TMT-A	−0.72 ± 0.62^***^	−0.21 ± 0.58^***^	0.94 ± 1.07	37.459 (<0.001)
TMT-B	−0.51 ± 0.27^***^	−0.23 ± 0.27^***^	0.75 ± 1.49	36.843 (<0.001)
Stroop words only	−0.72 ± 0.87^***^/^#^	−0.04 ± 0.69^***^	0.89 ± 0.75	32.855 (<0.001)
Stroop colors only	−0.63 ± 0.82^***^/^#^	−0.05 ± 0.72^***^	0.81 ± 0.83	22.399 (<0.001)
Animal naming test	−0.68 ± 0.60^***^/^#^	−0.14 ± 0.83^***^	0.96 ± 0.86	35.387 (<0.001)
COWAT	−0.64 ± 0.93^***^	−0.10 ± 1.02^*^	0.58 ± 0.67	12.535 (<0.001)
Stroop interference	−0.52 ± 0.93^***^	−0.13 ± 0.74^***^	0.81 ± 0.88	18.396 (<0.001)
Spatial processing	−0.41 ± 0.63^***^	−0.14 ± 0.46^***^	0.51 ± 0.48	22.713 (<0.001)
WAIS-RC	−0.81 ± 0.93^***^/^###^	0.03 ± 0.68^*^	0.70 ± 0.85	23.463 (<0.001)

*Data is presented as mean ± standard deviation. Adjusted age and education were employed as covariates. Bonferroni correction was used for post-hoc comparisons. Raw scores were converted to normalized Z-scores. BPRS, Brief Psychiatric Rating Scale; SANS, Scale for the Assessment of Negative Symptoms; SAPS, Scale for Assessment of Positive Symptoms. TMT-A/B, Trail Making Test-A/B; COWAT, Controlled Oral Word Association Test; WAIS-RC, Wechsler adult intelligence scale-Chinese Revision.^#^indicates the comparison between DS patients and NDS patients*.

# *p < 0.05*,

### *p < 0.001*.

* indicates the comparison between patients and HC. ^*^p < 0.05,

*** *p < 0.001*.

### Altered FC of NAcc Network Between DS, NDS, and HCs

**Figure 1:**
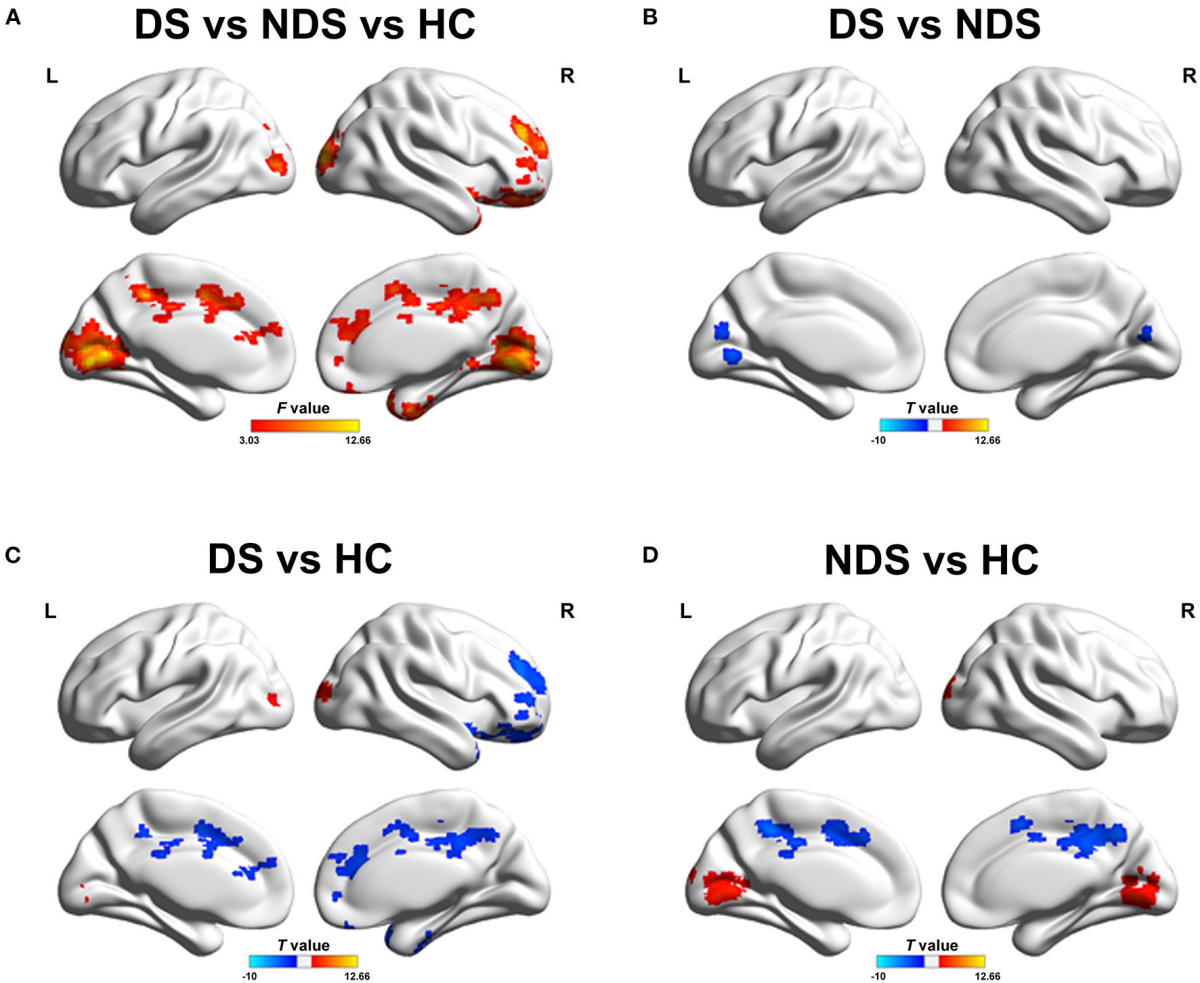
**(A)** Group comparisons of FC in left NAcc network among DS, NDS, and HC. The red regions represent significantly different regions. **(B)** Group analyses of the FC in left NAcc network between DS and NDS. The blue areas denote the regions where DS had lower FC compared to NDS. **(C,D)** Group analyses of the FC in left NAcc network between patients and HC groups. The blue areas indicate the regions where patients had lower FC compared to HC; the red areas signify the regions where patients had greater FC compared to HC. The significant threshold was set at *p* ≤ 0.05 after 3dClustSim corrected (voxel *p* ≤ 0.05, cluster size ≥ 200). FC, Functional Connectivity; NAcc, Nucleus accumbens; DS, Deficit Schizophrenia; NDS, Non-deficit Schizophrenia; HC, Healthy Controls.

**Figure 2:**
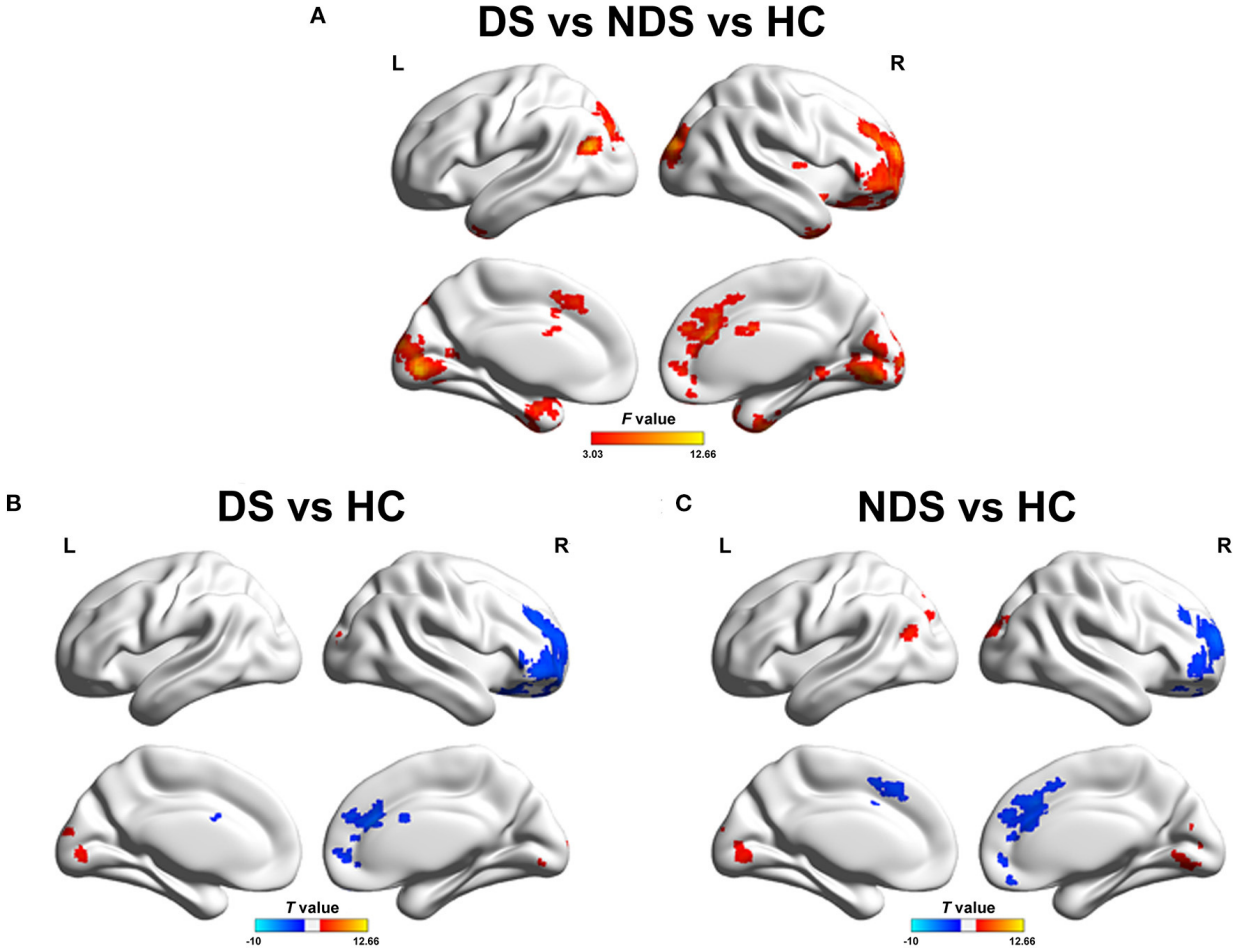
**(A)** Group comparisons of FC in right NAcc network among DS, NDS, and HC. The red areas denote significant different regions. **(B,C)** Group analyses of the FC in right NAcc network between patients and HC groups. The blue regions indicate the regions where patients had lower FC compared to HC; the red areas show the regions where patients had greater FC compared to HC. The significant threshold was set at *p* ≤ 0.05 after 3dClustSim corrected (voxel *p* ≤ 0.05, cluster size ≥ 200). FC, Functional Connectivity; NAcc, Nucleus accumbens; DS, Deficit Schizophrenia; NDS, Non-Deficit Schizophrenia; HC, Healthy Controls.

The analysis of altered FC in the NAcc network among DS patients, NDS patients, and HCs are shown in Figures 1, 2 and Tables 3, 4. Within the LNAcc network, both DS and NDS patients demonstrated lower FC between the LNAcc and bilateral middle cingulate gyrus (MCG) and higher FC between the LNAcc and bilateral lingual gyrus (LING), compared to HCs (Figures 1C,D). In addition, within the DS group, the FC of LNAcc with the right superior temporal gyrus (RSTG) and right middle frontal gyrus (RMFG) were lower, in contrast to the HC group (Figure 1C). Compared to NDS patients, the FC between the LNAcc and bilateral calcarine gyrus (CAL) in DS patients was significantly reduced (Figure 1B). In the RNAcc network, both the DS and NDS groups had decreased FC between RNAcc and RMFG, as well as increased FC between RNAcc and LING, in comparison with HCs (Figures 2B,C).

**Table 3:** Altered regions of FC in the left NAcc network based on ROI analysis.

**Brain regions**	**Peak MNI coordinate**			***F/t***	**Cluster size**
	**x**	**y**	**z**		
ANCOVA (df_F_ = 2, 259)					
R superior temporal gyrus/superior frontal gyrus	15	45	−18	9.2967	791
B lingual gyrus/calcarine/posterior cingulate/middle occipital gyrus	12	−75	−3	12.6588	2,273
R superior frontal gyrus/middle frontal gyrus/anterior cingulate	24	63	21	12.4595	912
B middle cingulate/precuneus	−9	−24	33	9.6436	968
DS vs. NDS (df*_*T*_* = 147)					
B calcarine/lingual	−9	−72	0	−3.431	264
DS vs. HC (df*_*T*_* = 167)					
R superior temporal gyrus/superior frontal gyrus	27	33	−21	−4.0576	536
B lingual gyrus/middle occipital gyrus	27	−96	9	4.4196	837
R middle frontal gyrus/superior frontal gyrus/anterior cingulate	27	42	27	−4.4527	743
B middle cingulate/precuneus	−9	0	48	−3.6016	618
NDS vs. HC (df*_*T*_* = 202)					
B lingual gyrus/calcarine/middle occipital gyrus	9	−72	−3	4.7406	2,082
B middle cingulate/precuneus	−9	−24	33	−4.7199	874

*MNI, Montreal Neurological Institute. Hemisphere location: R, Right; B, Bilateral*.

**Table 4:** Altered regions of FC in the right NAcc network based on ROI analysis.

**Brain regions**	**Peak MNI coordinate**			***F/t***	**Cluster size**
	**x**	**y**	**z**		
ANCOVA (df_F_ = 2, 259)					
R inferior temporal gyrus/superior temporal gyrus	39	−9	−39	9.1279	487
L inferior temporal gyrus/superior temporal gyrus	−21	12	−30	7.6495	342
B lingual gyrus/superior occipital gyrus/middle occipital gyrus/middle frontal gyrus/calcarine/precuneus	24	−96	24	12.3861	1,843
R middle frontal gyrus/superior frontal gyrus/anterior cingulate/inferior frontal gyrus	9	30	21	11.171	1,933
DS vs. HC (df*_*T*_* = 167)					
R middle frontal gyrus/superior frontal gyrus/anterior cingulate	9	30	21	−4.481	1,133
B lingual gyrus/middle occipital gyrus/superior occipital gyrus	−6	−84	0	3.9964	811
NDS vs. HC (df*_*T*_* = 202)					
R middle frontal gyrus/superior frontal gyrus/anterior cingulate	24	63	21	−4.4886	937
B lingual gyrus/calcarine/superior occipital gyrus/middle occipital gyrus	24	−96	24	4.8597	1,608

*MNI, Montreal Neurological Institute. Hemisphere location: R, Right; L, Left; B, Bilateral*.

### Correlation Between FC of NAcc Network With Clinical and Neurocognitive Features

**Figure 3:**
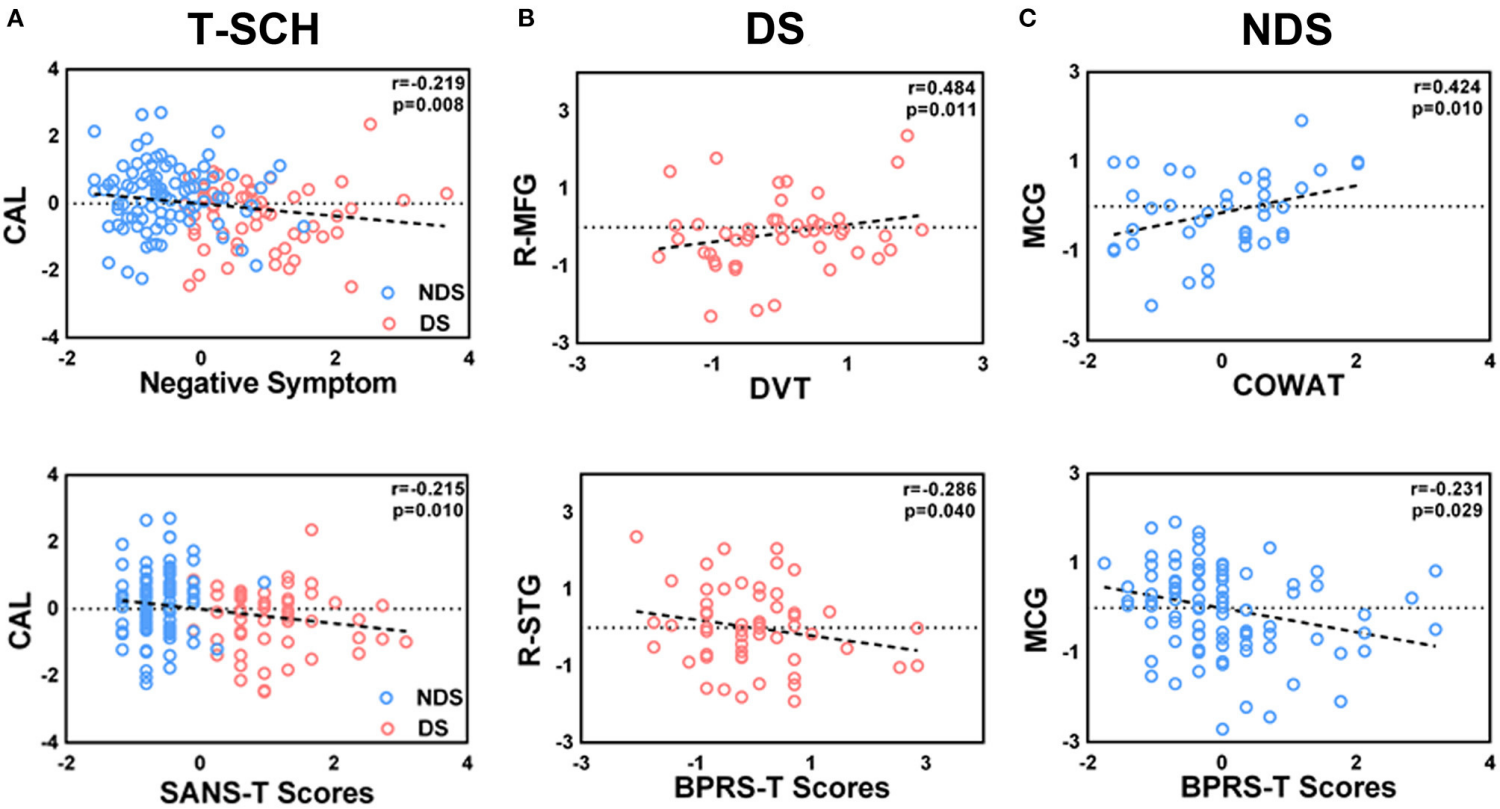
**(A–C)** Significant correlations between the altered FC of left NAcc network with clinical features and neurocognitive assessments in total patient group, DS group and NDS group. The significant threshold was set at *p* < 0.05 (uncorrected). FC, Functional Connectivity; T-SCH, Total Schizophrenia Patients; DS, Deficit Schizophrenia Patients; NDS, Non-Deficit Schizophrenia Patients; CAL, Calcarine; R-MFG, Right Middle Frontal Gyrus; R-STG, Right Superior Temporal Gyrus; MCG, Middle Cingulate Gyrus; DVT, Digit Vigilance Test; COWAT, Controlled Oral Word Association Test; SANS-T, Scale for the Assessment of Negative Symptoms; BPRS-T, Brief Psychiatric Rating Scale.

**Figure 4:**
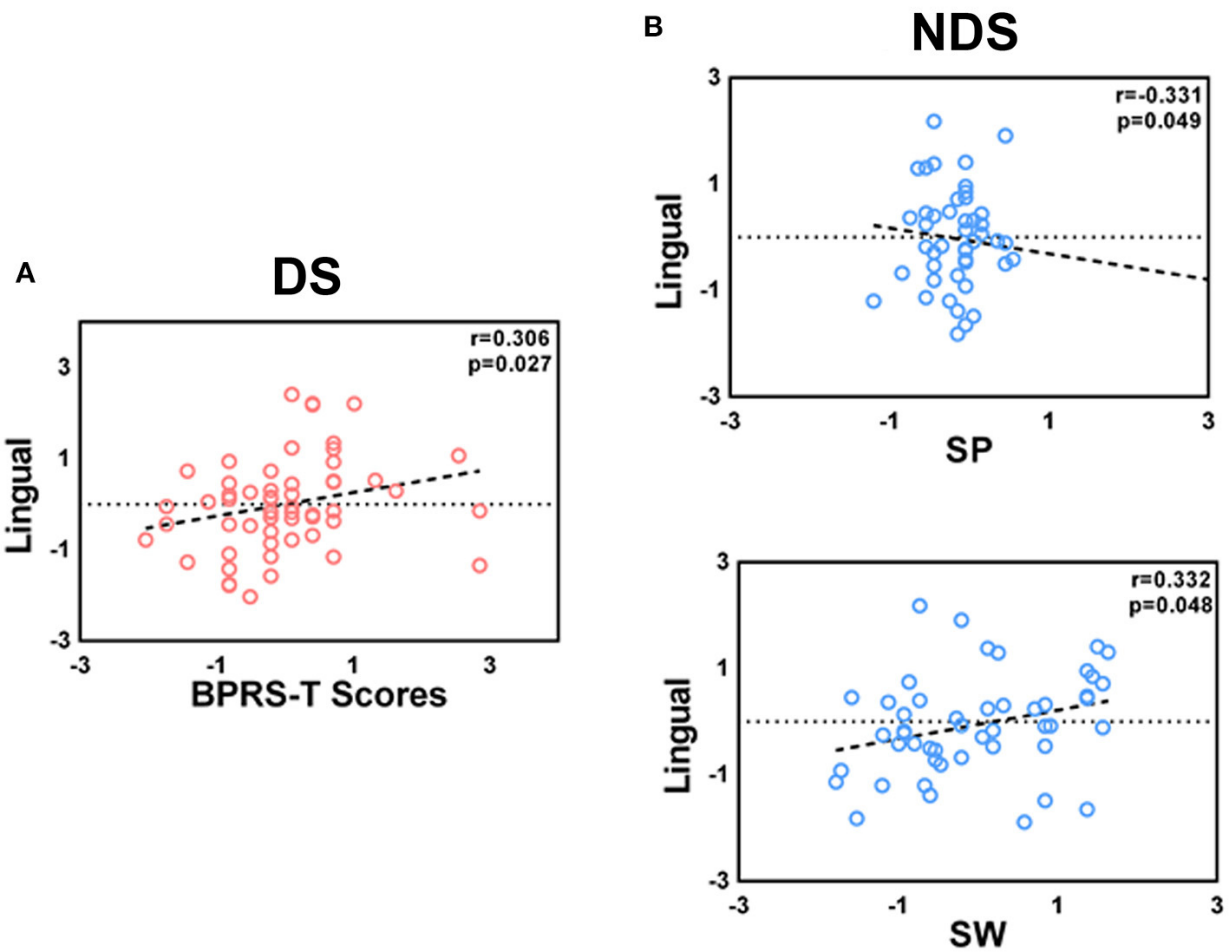
**(A,B)** Correlations between the altered FC of right NAcc network with clinical features and neurocognitive assessments in DS and NDS patients. The significant threshold was set at *p* < 0.05 (uncorrected). DS, Deficit Schizophrenia; NDS, Non-deficit Schizophrenia; BPRS-T, Brief Psychiatric Rating Scale; SP, Spatial processing; SW, Stroop words only.

Partial correlation analysis is described in Figures 3, 4. Among all patients, the FC value of LNAcc-CAL was significantly and negatively correlated to the negative syndrome subscale score of BPRS and total score of SANS (*r* = −0.219, *p* = 0.008, uncorrected; *r* = −0.215, *p* = 0.01, uncorrected; respectively). For the DS group, FC values of LNAcc-RSTG and RNAcc-LING were negatively and positively correlated to the total score of BPRS, respectively (*r* = −0.286, *p* = 0.040, uncorrected; *r* = 0.306, *p* = 0.027, uncorrected; respectively). In addition, the FC value of LNAcc-RMFG in DS patients was significantly and positively correlated to DVT score (*r* = 0.484, *p* = 0.011, uncorrected). Within the NDS group, the FC value of LNAcc-MCG was negatively correlated to the total score of BPRS, and positively correlated to COWAT score (*r* = −0.231, *p* = 0.029, uncorrected; *r* = 0.424, *p* = 0.01, uncorrected; respectively). Furthermore, the FC value of RNAcc-LING in NDS patients was negatively correlated to spatial processing test score, and positively correlated to Stroop words test score (*r* = −0.331, *p* = 0.049, uncorrected; *r* = 0.332, *p* = 0.048, uncorrected; respectively).

## Discussion

Our findings demonstrated that there were altered FC of the NAcc network in both DS and NDS patients. In addition, negative symptoms and neurocognition were significantly correlated. The main results of the present study include convergent altered FC of BNAcc with MCG, RMFG, and LING in both DS and NDS patients as well as lower divergent FC of LNAcc with CAL in DS patients. The relationship between FC of LNAcc-CAL and scale score of negative symptoms might indicate the potential pathogenesis of negative symptoms in schizophrenia. To our best knowledge, this is the first study to report altered patterns of FC within the NAcc network and their relationships with negative symptoms and neurocognition in patients with DS and NDS.

Our results indicated that the individual FC of BNAcc with MCG and RMFG were lower in DS and NDS patients compared to HCs. The MCG, comprising the anterior and posterior area, plays a role in both the salience network (SN) and sensorimotor network (SMN). MCG was involved in cognitive control functioning, sensorimotor integration and motor control (30). Previous studies have reported that a reduction in the gray matter volumes of MCG in schizophrenia patients (31) might be involved in an impaired functional role within the network. Consequently, the decreased FC between LNAcc and MCG observed in the present study might represent a functional disconnection between the reward network, SN and SMN in schizophrenia. MFG is comprised of a part of the dorsal lateral pre-frontal cortex (DLPFC) which is involved in working memory and executive cognitive functions (32, 33). In this study, the decreased FC between RNAcc and RMFG might potentially be the cause of emotional and cognitive dysregulations in schizophrenia patients (34). Furthermore, the reduced FC of the left and right NAcc network with extensive regions might indicate increased symptoms severity of schizophrenia. In the current study, the FC between BNAcc and LING was higher in both DS and NDS patients. The LING, which is located in the medial occipital lobe, is involved in visual processing and several cognitive functions (35, 36). A previous longitudinal study reported a significant increase of gray matter volumes within the bilateral lingual gyrus in schizophrenia. The authors also found that the increased volume of the left lingual gyrus was inversely related to functional outcomes (37). Therefore, it can be speculated that the structural alteration of the lingual gyrus observed in the present study might be involved in the hyperactive FC between BNAcc and LING and could contribute to the poor functional outcomes of schizophrenia patients. However, the aberrant increase in FC of BNAcc-LING in both DS and NDS needs to be further researched and elucidated.

Our findings additionally demonstrated that all the divergent altered regions in DS patients, such as the RSTG and RMFG, showed decreased FC with LNAcc. These results are consistent with a previous study which reported a decreased gray matter volume of LNAcc in DS patients (24). The divergent altered regions (i.e., RSTG and RMFG) observed in our study might result in the specific characteristics exhibited by DS patients. The STG is located in the primary auditory cortex and processes incoming auditory information (38). Most studies have reported reduced STG volumes in schizophrenia, compared to healthy subjects (39, 40). The STG has been shown to participate in the pathogenesis of both auditory and language processing dysfunctions, including auditory hallucinations and delusion (41, 42). The lower FC between LNAcc and RSTG might possibly reduce the strength or sensitivity of inputted auditory information, and further affects the development of clinical symptoms in DS patients. Notably, our study demonstrated differential FC of LNAcc-CAL between the two patient subgroups, which might represent a core region of altered FC within the LNAcc network. The CAL, located in the middle occipital gyrus, is involved in the primary visual cortex. Prior studies have reported the presence of reduced gray matter volume and structural connections of CAL in schizophrenia compared to HCs (43, 44). While there is currently no anatomical evidence which supports a connection between the NAcc and CAL, it was previously demonstrated that CAL had a lower functional stability in schizophrenia (45). In the present study, the abnormality of CAL might contribute to the altered FC of CAL with LNAcc in DS patients. Subsequently, both the structural and functional connections between NAcc and CAL need to be further investigated.

The current study revealed that the FC value of LNAcc-CAL was significantly negatively correlated with negative symptoms in all patients with schizophrenia. Moreover, the relationship between negative symptoms and altered FC within the NAcc network indicated specific and distinct clinical and neuroimaging features in DS patients compared to NDS patients. It was previously reported that aberrant structural alterations of NAcc were involved in the pathogenesis of negative symptoms in schizophrenia (11, 46). Our study showed that altered FC of NAcc correlated with negative symptoms on the aspect of functional integration. Furthermore, our findings described the relationships between severity of symptoms, cognitive impairments and the FC of NAcc network, which provides novel perspectives in the pathogenesis of DS and NDS. Interestingly, in this study, altered FC of LNAcc-MCG and RNAcc-Ling within the NAcc network was associated with severity of symptoms and neurocognitive deficits in both DS and NDS patients. These results suggest a common mechanism which relate to symptoms or cognitive impairments in schizophrenia (47). However, given that correlation analyses have limited statistical power and there is a possibility of false positive findings, our results should be corroborated by future studies.

There are several limitations in the present study that should be considered. Firstly, patients enrolled in this study were all chronic male inpatients since the purpose was to increase the homogeneity of participants. Future studies should consider collecting larger datasets that also include female subjects so as to explore gender differences and to improve the statistical power. Secondly, the recruited patients were under stable and long-term antipsychotic medication treatment. The impact of antipsychotic medication on FC in the NAcc network and/or in cognitive functions should be considered and excluded (10). In our study, we found no significant association between antipsychotic dosage and neuroimaging/cognition metrics. Finally, the current study should be considered as an exploratory analysis where the *p* < 0.05 (after correction) level was used for statistical validity. Subsequent studies could further increase the sample size and perform stricter statistical correction in order to explore alterations in the structure or function of the brain in DS and NDS patients.

In summary, our findings revealed the convergent and divergent altered FC of NAcc network in DS and NDS patients. The aberrant FC of BNAcc with RMFG, MCG, and LING regions might represent a commonality between the two subgroups of patients. The divergent altered FC of LNAcc-CAL could potentially be used as a characteristic feature for distinguishing DS from NDS patients. Furthermore, the correlation between altered FC of LNAcc-CAL might indicate the functional alteration of NAcc related to negative symptoms in schizophrenia.

## Data Availability Statement

The raw data supporting the conclusions of this article will be made available by the authors, without undue reservation.

## Ethics Statement

The studies involving human participants were reviewed and approved by the Institutional Ethical Committee for clinical research of ZhongDa Hospital Affiliated to Southeast University (2013ZDSYLL52.0). The patients/participants provided their written informed consent to participate in this study.

## Author Contributions

XZ and CZha designed and organized the research. XT and HZ collected the imaging and cognitive data. JC, CZho, and CX analyzed the data. NA, CZho, and FZ wrote and revised the manuscript. All authors contributed to the article and approved the submitted version.

## Funding

This work was supported by the National Natural Science Foundation of China (NSFC) (Nos. 81971255, 82101572), the National Key Research and Development Program of China (No. 2018YFC1314303), Social Development Foundation of Jiangsu Province, China (No. BE2019610), Basic Research Project of Frontier Technology in Jiangsu Province (BK20192004D), and Jiangsu Provincial Medical Talent project (ZDRCA2016075).

## Conflict of Interest

The authors declare that the research was conducted in the absence of any commercial or financial relationships that could be construed as a potential conflict of interest.

## Publisher's Note

All claims expressed in this article are solely those of the authors and do not necessarily represent those of their affiliated organizations, or those of the publisher, the editors and the reviewers. Any product that may be evaluated in this article, or claim that may be made by its manufacturer, is not guaranteed or endorsed by the publisher.

## References

[1] Li Z Zhang CY Huang J Wang Y Yan C Li K. Improving motivation through real-time fMRI-based self-regulation of the nucleus accumbens. Neuropsychology. (2018) 32:764–76. 10.1037/neu0000425 30047755

[2] Lum JS Millard SJ Huang XF Ooi L Newell KA. A postmortem analysis of NMDA ionotropic and group 1 metabotropic glutamate receptors in the nucleus accumbens in schizophrenia. J Psychiatry Neurosci. (2018) 43:102–10. 10.1503/jpn.170077 29481317 PMC5837882

[3] Nielsen MO Rostrup E Broberg BV Wulff S Glenthoj B. Negative symptoms and reward disturbances in schizophrenia before and after antipsychotic monotherapy. Clin EEG Neurosci. (2018) 49:36–45. 10.1177/1550059417744120 29145751

[4] Mavridis I. The role of the nucleus accumbens in psychiatric disorders]. Psychiatriki. (2015) 25:282–94. 26709994

[5] Robison AJ Thakkar KN Diwadkar VA. Cognition and reward circuits in schizophrenia: synergistic, not separate. Biol Psychiatry. (2020) 87:204–14. 10.1016/j.biopsych.2019.09.021 31733788 PMC6946864

[6] Bois C Levita L Ripp I Owens DC Johnstone EC Whalley HC. Hippocampal, amygdala and nucleus accumbens volume in first-episode schizophrenia patients and individuals at high familial risk: a cross-sectional comparison. Schizophr Res. (2015) 165:45–51. 10.1016/j.schres.2015.03.024 25864953

[7] Ebdrup BH Glenthoj B Rasmussen H Aggernaes B Langkilde AR Paulson OB. Hippocampal and caudate volume reductions in antipsychotic-naive first-episode schizophrenia. J Psychiatry Neurosci. (2010) 35:95–104. 10.1503/jpn.090049 20184807 PMC2834791

[8] Okada N Fukunaga M Yamashita F Koshiyama D Yamamori H Ohi K. Abnormal asymmetries in subcortical brain volume in schizophrenia. Mol Psychiatry. (2016) 21:1460–6. 10.1038/mp.2015.209 26782053 PMC5030462

[9] van Erp TG Hibar DP Rasmussen JM Glahn DC Pearlson GD Andreassen OA. Subcortical brain volume abnormalities in 2028 individuals with schizophrenia and 2540 healthy controls via the ENIGMA consortium. Mol Psychiatry. (2016) 21:585. 10.1038/mp.2015.118 26283641 PMC5751698

[10] Boonstra G van Haren NE Schnack HG Cahn W Burger H Boersma M. Brain volume changes after withdrawal of atypical antipsychotics in patients with first-episode schizophrenia. J Clin Psychopharmacol. (2011) 31:146–53. 10.1097/JCP.0b013e31820e3f58 21346618

[11] Lee JS Chun JW Kang JI Kang DI Park HJ Kim JJ. Hippocampus and nucleus accumbens activity during neutral word recognition related to trait physical anhedonia in patients with schizophrenia: an fMRI study. Psychiatry Res. (2012) 203:46–53. 10.1016/j.pscychresns.2011.09.004 22867952

[12] Rolland B Amad A Poulet E Bordet R Vignaud A Bation R. Resting-state functional connectivity of the nucleus accumbens in auditory and visual hallucinations in schizophrenia. Schizophr Bull. (2015) 41:291–9. 10.1093/schbul/sbu097 25053649 PMC4266295

[13] Carpenter WT Jr Heinrichs DW Wagman AM. Deficit and nondeficit forms of schizophrenia: the concept. Am J Psychiatry. (1988) 145:578–83. 10.1176/ajp.145.5.578 3358462

[14] Blanchard JJ Horan WP Collins LM. Examining the latent structure of negative symptoms: is there a distinct subtype of negative symptom schizophrenia? Schizophr Res. (2005) 77:151–65. 10.1016/j.schres.2005.03.022 15916881

[15] Ahmed AO Strauss GP Buchanan RW Kirkpatrick B Carpenter WT. Are negative symptoms dimensional or categorical? Detection and validation of deficit schizophrenia with taxometric and latent variable mixture models. Schizophr Bull. (2015) 41:879–91. 10.1093/schbul/sbu163 25399026 PMC4466177

[16] Yu M Dai Z Tang X Wang X Zhang X Sha W. Convergence and divergence of brain network dysfunction in deficit and non-deficit schizophrenia. Schizophr Bull. (2017) 43:1315–28. 10.1093/schbul/sbx014 29036672 PMC5737538

[17] Zhou C Yu M Tang X Wang X Zhang X Zhang X. Convergent and divergent altered patterns of default mode network in deficit and non-deficit schizophrenia. Prog Neuropsychopharmacol Biol Psychiatry. (2019) 89:427–34. 10.1016/j.pnpbp.2018.10.012 30367960

[18] Kirkpatrick B Galderisi S. Deficit schizophrenia: an update. World Psychiatry. (2008) 7:143–7. 10.1002/j.2051-5545.2008.tb00181.x 18836581 PMC2559917

[19] Galderisi S Maj M. Deficit schizophrenia: an overview of clinical, biological and treatment aspects. Eur Psychiatry. (2009) 24:493–500. 10.1016/j.eurpsy.2009.03.001 19553087

[20] Kirkpatrick B. Recognizing primary vs secondary negative symptoms and apathy vs expression domains. J Clin Psychiatry. (2014) 75:e09. 10.4088/JCP.13049tx3c 24813410

[21] Xie T Zhang X Tang X Zhang H Yu M Gong G. Mapping convergent and divergent cortical thinning patterns in patients with deficit and nondeficit schizophrenia. Schizophr Bull. (2019) 45:211–21. 10.1093/schbul/sbx178 29272543 PMC6293229

[22] Zhou C Tang X You W Wang X Zhang X Zhang X. Altered patterns of the fractional amplitude of low-frequency fluctuation and functional connectivity between deficit and non-deficit schizophrenia. Front Psychiatry. (2019) 10:680. 10.3389/fpsyt.2019.00680 31572248 PMC6754073

[23] Gao J Tang X Wang C Yu M Sha W Wang X. Aberrant cerebellar neural activity and cerebro-cerebellar functional connectivity involving executive dysfunction in schizophrenia with primary negative symptoms. Brain Imaging Behav. (2020) 14:869–80. 10.1007/s11682-018-0032-9 30612342

[24] De Rossi P Dacquino C Piras F Caltagirone C Spalletta G. Left nucleus accumbens atrophy in deficit schizophrenia: a preliminary study. Psychiatry Res Neuroimaging. (2016) 254:48–55. 10.1016/j.pscychresns.2016.06.004 27322868

[25] First MB William Janet BW Spitzer RL Gibbon M. Structured Clinical Interview for DSM-IV Axis I Disorders: Non-patient Edition (SCID-NP). New York, NY: Biometrics Research Department (1996).

[26] Wang X Yao S Kirkpatrick B Shi C Yi J. Psychopathology and neuropsychological impairments in deficit and nondeficit schizophrenia of Chinese origin. Psychiatry Res. (2008) 158:195–205. 10.1016/j.psychres.2006.09.007 18243336

[27] Cohen AS Saperstein AM Gold JM Kirkpatrick B Carpenter WT Jr Buchanan RW. Neuropsychology of the deficit syndrome: new data and meta-analysis of findings to date. Schizophr Bull. (2007) 33:1201–12. 10.1093/schbul/sbl066 17159230 PMC2632354

[28] Jenkinson M Bannister P Brady M Smith S. Improved optimization for the robust and accurate linear registration and motion correction of brain images. Neuroimage. (2002) 17:825–41. 10.1016/s1053-8119(02)91132-8 12377157

[29] Maldjian JA Laurienti PJ Kraft RA Burdette JH. An automated method for neuroanatomic and cytoarchitectonic atlas-based interrogation of fMRI data sets. Neuroimage. (2003) 19:1233–9. 10.1016/s1053-8119(03)00169-1 12880848

[30] Wang D Zhou Y Zhuo C Qin W Zhu J Liu H. Altered functional connectivity of the cingulate subregions in schizophrenia. Transl Psychiatry. (2015) 5:e575. 10.1038/tp.2015.69 26035059 PMC4490280

[31] Li H Tang J Chen L Liao Y Zhou B He Y. Reduced middle cingulate gyrus volume in late-onset schizophrenia in a Chinese Han population: a voxel-based structural MRI study. Neurosci Bull. (2015) 31:626–7. 10.1007/s12264-015-1525-1 25956581 PMC5563674

[32] van Veelen NM Vink M Ramsey NF Kahn RS. Left dorsolateral prefrontal cortex dysfunction in medication-naive schizophrenia. Schizophr Res. (2010) 123:22–9. 10.1016/j.schres.2010.07.004 20724113

[33] Barbey AK Koenigs M Grafman J. Dorsolateral prefrontal contributions to human working memory. Cortex. (2013) 49:1195–205. 10.1016/j.cortex.2012.05.022 22789779 PMC3495093

[34] Anticevic A Schleifer C Youngsun TC. Emotional and cognitive dysregulation in schizophrenia and depression: understanding common and distinct behavioral and neural mechanisms. Dialogues Clin Neurosci. (2015) 17:421–34. 10.31887/DCNS.2015.17.4/aanticevic 26869843 PMC4734880

[35] Stephan-Otto C Siddi S Cuevas Esteban J Senior C Garcia-Alvarez R Cambra-Marti MR. Neural activity during object perception in schizophrenia patients is associated with illness duration and affective symptoms. Schizophr Res. (2016) 175:27–34. 10.1016/j.schres.2016.04.020 27130563

[36] Fan FM Xiang H Wen Y Zhao YL Zhu XL Wang YH. Brain abnormalities in different phases of working memory in schizophrenia: an integrative multi-modal MRI study. J Nerv Ment Dis. (2019) 207:760–7. 10.1097/NMD.0000000000001001 31465311

[37] Mane A Falcon C Mateos JJ Fernandez-Egea E Horga G Lomena F. Progressive gray matter changes in first episode schizophrenia: a 4-year longitudinal magnetic resonance study using VBM. Schizophr Res. (2009) 114:136–43. 10.1016/j.schres.2009.07.014 19683418

[38] Morosan P Rademacher J Schleicher A Amunts K Schormann T Zilles K. Human primary auditory cortex: cytoarchitectonic subdivisions and mapping into a spatial reference system. Neuroimage. (2001) 13:684–701. 10.1006/nimg.2000.0715 11305897

[39] Takahashi T Wood SJ Yung AR Walterfang M Phillips LJ Soulsby B. Superior temporal gyrus volume in antipsychotic-naive people at risk of psychosis. Br J Psychiatry. (2010) 196:206–11. 10.1192/bjp.bp.109.069732 20194543

[40] Ohi K Matsuda Y Shimada T Yasuyama T Oshima K Sawai K. Structural alterations of the superior temporal gyrus in schizophrenia: detailed subregional differences. Eur Psychiatry. (2016) 35:25–31. 10.1016/j.eurpsy.2016.02.002 27061374

[41] Shenton ME Dickey CC Frumin M McCarley RW. A review of MRI findings in schizophrenia. Schizophr Res. (2001) 49:1–52. 10.1016/s0920-9964(01)00163-3 11343862 PMC2812015

[42] Allen P Laroi F McGuire PK Aleman A. The hallucinating brain: a review of structural and functional neuroimaging studies of hallucinations. Neurosci Biobehav Rev. (2008) 32:175–91. 10.1016/j.neubiorev.2007.07.012 17884165

[43] Maggioni E Crespo-Facorro B Nenadic I Benedetti F Gaser C Sauer H. Common and distinct structural features of schizophrenia and bipolar disorder: the European Network on Psychosis, Affective disorders and Cognitive Trajectory (ENPACT) study. PLoS ONE. (2017) 12:e0188000. 10.1371/journal.pone.0188000 29136642 PMC5685634

[44] Qin J Sui J Ni H Wang S Zhang F Zhou Z. The shared and distinct white matter networks between drug-naive patients with obsessive-compulsive disorder and schizophrenia. Front Neurosci. (2019) 13:96. 10.3389/fnins.2019.00096 30846924 PMC6393388

[45] Zhu J Zhang S Cai H Wang C Yu Y. Common and distinct functional stability abnormalities across three major psychiatric disorders. Neuroimage Clin. (2020) 27:102352. 10.1016/j.nicl.2020.102352 32721869 PMC7393318

[46] Bracht T Horn H Strik W Federspiel A Razavi N Stegmayer K. White matter pathway organization of the reward system is related to positive and negative symptoms in schizophrenia. Schizophr Res. (2014) 153:136–42. 10.1016/j.schres.2014.01.015 24485586

[47] Harvey PD Koren D Reichenberg A Bowie CR. Negative symptoms and cognitive deficits: what is the nature of their relationship? Schizophr Bull. (2006) 32:250–8. 10.1093/schbul/sbj011 16221995 PMC2632205

